# Substrate Oxide Layer Thickness Optimization for a Dual-Width Plasmonic Grating for Surface-Enhanced Raman Spectroscopy (SERS) Biosensor Applications

**DOI:** 10.3390/s17071530

**Published:** 2017-06-30

**Authors:** Stephen J. Bauman, Zachary T. Brawley, Ahmad A. Darweesh, Joseph B. Herzog

**Affiliations:** 1 Microelectronics-Photonics Graduate Program, 731 W. Dickson St., University of Arkansas, Fayetteville, Arkansas, AR 72701, USA; sjbauman@email.uark.edu (S.J.B.); aadarwee@email.uark.edu (A.A.D.); 2 Department of Physics, 825 W. Dickson St., University of Arkansas, Fayetteville, Arkansas, AR 72701, USA; ztbrawle@email.uark.edu

**Keywords:** Raman spectroscopy, SERS, plasmonics, nano-optics, computational electromagnetics, plasmonic grating, nanogap

## Abstract

This work investigates a new design for a plasmonic SERS biosensor via computational electromagnetic models. It utilizes a dual-width plasmonic grating design, which has two different metallic widths per grating period. These types of plasmonic gratings have shown larger optical enhancement than standard single-width gratings. The new structures have additional increased enhancement when the spacing between the metal decreases to sub-10 nm dimensions. This work integrates an oxide layer to improve the enhancement even further by carefully studying the effects of the substrate oxide thickness on the enhancement and reports ideal substrate parameters. The combined effects of varying the substrate and the grating geometry are studied to fully optimize the device’s enhancement for SERS biosensing and other plasmonic applications. The work reports the ideal widths and substrate thickness for both a standard and a dual-width plasmonic grating SERS biosensor. The ideal geometry, comprising a dual-width grating structure atop an optimal SiO_2_ layer thickness, improves the enhancement by 800%, as compared to non-optimized structures with a single-width grating and a non-optimal oxide thickness.

## 1. Introduction

The use of plasmonic nanostructures in sensing applications has been demonstrated via an increasing number of methods over the past decades, as nanoscience and technology have continued to improve. Better imaging resolution [1,2,3], device fabrication [4,5,6], and other improvements have allowed for the development of single-molecule spectroscopy techniques [7,8,9], robust techniques capable of potential commercialization or mass production [10,11,12], and novel biomedical methods and technologies [8,13,14,15]. Different types of electromagnetic-radiation-based strategies are used to image, sense, detect, diagnose, and characterize various biological entities and processes [16,17,18].

For chemical and specifically biological sensing, Raman spectroscopy holds the benefit of high specificity over techniques like photoluminescence and fluorescence spectroscopy, which requires known tags to be assigned to the analytes for detection [9,19,20]. Raman spectroscopy makes use of the characteristic vibrational modes of analyte molecules upon exposure to an incident light to allow the user to detect the presence of specific chemical elements in a sample. Raman can be used with solid, liquid, and gas phase samples, a benefit that has allowed it to be applied to biological specimens [21,22]. Desirable applications of Raman spectroscopy include detection of harmful or beneficial elements in a biomedical environment [23,24,25], biosecurity applications (such as in airports) [26,27,28], and environmental safety concerns such as in the home and workplace [29,30,31,32,33]. These require high selectivity for accurate differentiation between molecular species and high sensitivity for the detection of trace levels of various elements, especially for detectors of dangerous chemicals. The main disadvantage of Raman spectroscopy is the low intensity of the signal, which leads to low sensitivity of detection. This difficulty of detection is exaggerated for lower concentrations of the desired analyte molecule. This has been a crucial issue to solve for the commercialization of handheld Raman spectroscopy technology, for sensing a variety of chemicals, sample types, and environments where concentrations may vary significantly [34,35,36,37]. Plasmonic devices can solve this issue.

Metallic and (in some cases) dielectric nanostructures, with features on the order of the wavelength of an incident optical signal, exhibit plasmon resonances that couple strongly to the incoming light [38,39,40,41]. Plasmonic resonances are collective oscillations of free electrons within a metal, and when the metallic structure is of the order of hundreds of nanometers or smaller, these oscillations can resonate strongly over the full geometry without rapidly decaying. Because plasmons are waves of charged particles, they produce an oscillatory electric field that interacts with other nearby fields. For certain nanostructure geometries and materials, and a given incoming light wavelength, the resonance can be quite strong. The result is the production of local plasmonic hot spots, or regions where the electric field strength is greatly increased over that of the incoming light [42,43,44,45]. Prior research has demonstrated that bringing two or more structures within separation distances of 10 nm can improve this effect nearly exponentially, resulting in significant increases in the local field enhancement [46,47,48,49,50,51].

Increasing the sensitivity of detection of the Raman signal is where plasmonics provide benefit to the technique, resulting in what is known as surface-enhanced Raman spectroscopy (SERS) [52,53,54]. SERS has been demonstrated as a highly selective technique for biosensing, capable of sensitivities down to single molecule detection [55,56,57]. An ideal nanoplasmonic SERS biosensor device should be tunable, via rational fabrication or post-fabrication tunability, to a specific wavelength (or multiple wavelengths) of light in order to significantly increase the strength of the analyte molecule’s Raman-scattered signal [53,54]. Improved fabrication capabilities in recent years have made possible new types of nanostructure geometries, some of which have been designed as SERS substrates for biological sensing applications [58,59,60,61].

Previous work has detailed the development of what has been referred to as the nanomasking fabrication technique, which allows for the creation of sub-10 nm gaps between metallic nanostructures with a large degree of control over the structure geometries [62]. The technique can produce a large area of nanogap structures in two or less lithography steps, depending on the pattern desired, and is thus appealing for the development of a SERS biosensor.

Plasmonic gratings provide the added benefit of covering a large area of the substrate, so as to improve the signal in many places, simultaneously, and also increase the enhancement with plasmonic grating effects [63,64,65]. For a biosensor substrate, the presence of many plasmonic hot spots increases the likelihood of locating an analyte molecule within one of these field-enhancing regions, thus improving the probability of detecting the presence of the desired species [66]. As with other types of plasmonic enhancement devices, the ability to control the geometric and material parameters during fabrication is crucial to the ability to tune devices to specific light wavelengths. Different types of gratings and grating-like structures have been demonstrated experimentally for this purpose [67,68,69,70]. Prior work has studied what have been called dual-width or double-width plasmonic gratings for use in GaAs photodetector applications [71]. This theoretical work demonstrated how the dual-width geometry can provide additional control over the optimization of the device. These dual-width gratings contain two different nanostructure widths in each period, along the direction of periodicity. This differs from a standard (or single-width) grating, which contains a single Au width that repeats for each period. The theoretical benefit of this type of device, integrated with an optimal oxide layer thickness, has yet to be studied for use in SERS biosensor applications. The oxide layer can also be optimized to provide constructive interference with the light scattered by the plasmonic structure.

The nanomasking fabrication technique, as outlined by Bauman et al. [62], is capable of producing dual-width nanogap gratings. This method provides control over the sizes of the plasmonic structures, making it tunable to the desired wavelength for conducting SERS. The high degree of geometric control allows the user to separately design the two widths of the grating, greatly increasing this tunability. The ability to simultaneously fabricate many sub-10 nm gaps between the structures is also beneficial to the development of a biosensing substrate with many hot spots. The work described herein details the computational study of dual-width plasmonic gratings for use as SERS substrates for biosensing applications.

Also controllable during the fabrication process, the thickness of the substrate oxide layer is found to play a significant role in producing the greatest signal enhancement, and is therefore worth studying to optimize the biosensing device. The geometric parameters of the grating are analyzed along with the oxide thickness on a SiO_2_/Si substrate, to optimize the device for maximum light enhancement within the gap region. Therefore, the proposed dual-width grating device is desirable for SERS due to its high tunability, the presence of many plasmonic hot spots, and the degree of enhancement capable of being produced by a fully optimized structure. Previous works report comparisons of plasmonic structures for different surrounding dielectric media [72,73,74]. The present work additionally shows that for a given substrate with a top oxide, it can be valuable to optimize this layer in addition to tailoring the plasmonic structures themselves, to maximize the enhancement factor. While some have shown improvement using Au substrates [75], we demonstrate improvement via the cheaper, more commonly used oxide layer that is easily grown on various substrates. The current work describes the combined optimization of the newly developed dual-width plasmonic grating geometry with oxide thickness on a Si/SiO_2_ substrate.

## 2. Materials and Methods 

**Figure 1:**
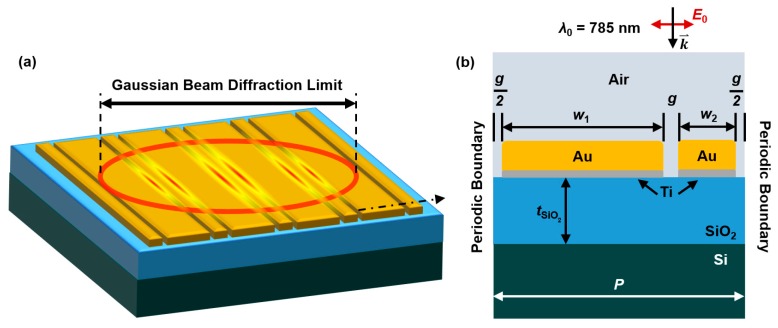
Sketch of the dual-width plasmonic grating structure, on a Si substrate containing a layer of SiO_2_, with finite thickness: (**a**) shows a 3D representation of the structure and the generation of plasmonic hot spots within the area of a diffraction-limited Gaussian beam; (**b**) shows a 2D cross-section of the structure in (**a**) as designed in the computational models. The structure widths (*w*_1_ and *w*_2_), spacing width (*g*), period (*P*), and SiO_2_ thickness (*t*_SiO2_) are labeled, along with the incident wavelength (*λ*_0_), propagation direction (*k*), and electric field polarization (*E*_0_).

This work utilized a two-dimensional finite element model to calculate the electric field at each mesh point by simulating light with a 785 nm wavelength incident on the top surface of a plasmonic grating structure and underlying substrate [76]. Figure 1 displays sketches of the simulated entities. Figure 1a is a three-dimensional sketch of a few periods of the dual-width Au grating structure on a SiO_2_/Si substrate with a rendering of the generation of plasmonic hot spots in the nanogaps within the area of a diffraction-limited laser beam spot. Figure 1b is the computational model geometry which is a cross-sectional of a single period of the structure, shown in Figure 1a. The model was created, as shown in Figure 1b, with material properties for air, Au, Ti, SiO_2_, and Si assigned to the applicable regions. The Ti adhesion layer is visible in Figure 1b, along with geometric and other variables (used in the model listed for clarity).

In this work, the incident field strength and polarization direction (*E*_0_) were held constant, along with the propagation direction (*k*) and incident light wavelength (*λ*_0_ = 785 nm). The width of the spacing between structures and the thickness of the Au and Ti layers, having been studied for optimization in previous work and having limits due to fabrication parameters, were held constant [77,78,79]. The spacing (or gap) width, *g*, was held constant at 5 nm, the Au thickness at 15 nm, and the Ti thickness at 1 nm. The variables for this study include the two grating structure widths, *w*_1_ and *w*_2_, and the thickness of the SiO_2_ layer, *t*_SiO2_. Varying the structure widths also changes the period of the grating (*P*), and thus the width of the simulation space. In all cases, *P* = *w*_1_ + *w*_2_ + 2*g*. The simulation space contains only one period of the grating, so the horizontal boundaries were made periodic to simulate an infinitely repeating dual-structure grating.

This study optimizes the substrate oxide thickness with the dual-width geometry, to produce the maximum increase in electric field strength within the nanogap (the hot spot region of the sensor). Thus, the calculations were comprised of combinations of different ranges of sweeps for *w*_1_, *w*_2_, and *t*_SiO2_. The optical enhancement is defined as the square of the ratio between the local electric field and the incident field strength, (*E*/*E*_0_)^2^. Reported optical enhancement values were taken from an integration area surrounding the gap and the hot spot. The average enhancement was calculated within this area for each geometric situation reported, and reported as an average of (*E*/*E*_0_)^2^ in the plots within the following figures. The size of this area remained constant as a function of all the variables. 

## 3. Results

The solution to the computation is the electric field distribution (EFD) of the grating cross-section. In the following subsections, the substrate oxide thickness and structure widths were optimized to produce the greatest local electric field within the gap region, which is where analyte signals will be most greatly enhanced in a sensing application.

### 3.1. Substrate Thickness Optimization

**Figure 2:**
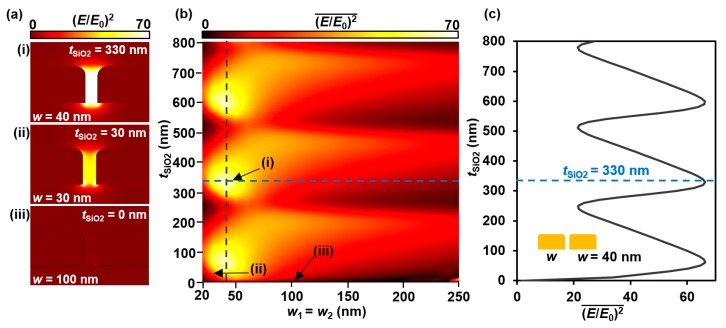
Calculated results when varying *t*_SiO2_ and *w* simultaneously: (**a**) representative enhancement distribution of three geometry combinations: (i) maximum value at *w* = 40 nm and *t*_SiO2_ = 330 nm, (ii) median value at *w* = 30 nm and *t*_SiO2_ = 30 nm, and (iii) minimum value at *w* = 100 nm and *t*_SiO2_ = 0 nm; (**b**) color plot showing average enhancement versus gold width *w* and oxide thickness *t*_SiO2_. The gray vertical dotted line traces the optimal *w* and the blue line tracks along one instance of optimal oxide thickness; and (**c**) plot of *t*_SiO2_ versus average optical enhancement at *w* = 40 nm, the optimal value for a single-width grating.

First, a standard single-width plasmonic grating was analyzed as a baseline and reference structure. Setting *w* = *w*_1_ = *w*_2_, the width was swept from 20 nm to 250 nm by 10 nm increments. In addition, *t*_SiO2_ was swept from 0 nm to 800 nm by 10 nm increments on each successive expansion of *w,* so that all possible combinations were analyzed. Enhancement distributions are shown in Figure 2a, and Figure 2b plots the average of (*E*/*E*_0_)^2^ vs. *w* and *t*_SiO2_.

**Figure 3:**
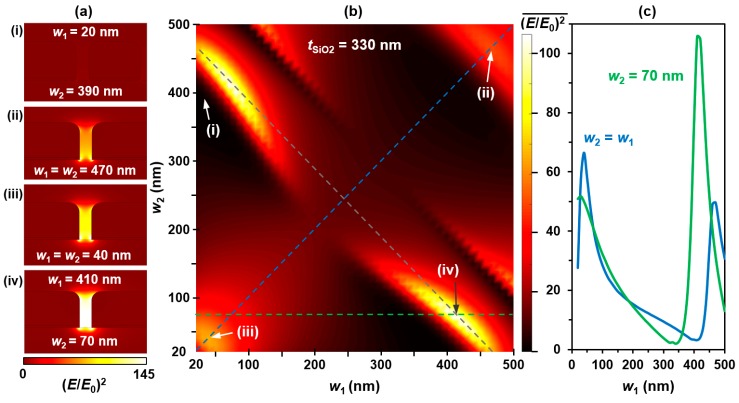
Calculated results when simultaneously varying *w*_1_ and *w*_2_ constant oxide thickness, *t*_SiO2_ = 330 nm: (**a**) representative enhancement distributions of four geometry combinations: (i) Minimum value at *w*_1_ = 20 nm and *w*_2_ = 390 nm, (ii) single-width secondary peak value at *w* = *w*_1_ *= w*_2_ = 470 nm, (iii) single-width primary peak value at *w* = 40 nm, and (iv) maximum value at *w*_1_ = 410 nm and *w*_2_ = 70 nm; (**b**) color plot showing average enhancement values for combinations of *w*_2_ and *w*_1_. Blue, gray, and green dashed lines correspond to single-width values, constant period, and constant *w*_2_ at peak enhancement, respectively; and (**c**) plot of (*E*/*E*_0_)^2^ versus *w*_1_ along the blue and green dashed lines in (**b**).

It was found that the average enhancement value is periodic in nature, as *t*_SiO2_ increases for a constant grating width. The vertical gray line in Figure 2b shows that the optimized structure width occurs at *w* = 40 nm, and the horizontal blue line shows the optimal SiO_2_ thickness, *t*_SiO2_ = 330 nm, which is the same data as is plotted along the blue diagonal line in Figure 3b. As *w* increases beyond the peak value, the average (*E*/*E*_0_)^2^ decreases. Figure 2c tracks along the gray vertical dashed line of Figure 2b, and shows the periodicity of the enhancement response as *t*_SiO2_ increases. This is due to the thin film interference effects of the oxide layer, as discussed further in Section 4. While the optimal oxide thickness was determined to be at *t*_SiO2_ = 330 nm, the enhancement here was only slightly larger than the enhancement at the other peaks. Therefore, for this particular single width structure with a 15 nm Au thickness and a 5 nm gap, and when holding the width to the optimal width of *w* = 40 nm, an optimal oxide thickness can be *t_m_* = *m* (270 nm) + 60 nm, where *m* = {1, 2, 3…}. 

The optimized geometry is found where the blue and gray lines in Figure 2b intersect. Figure 2a shows the enhancement distributions cross-section at the gap of three specific geometric combinations: (i) maximum (*E*/*E*_0_)^2^ and optimized geometry, (ii) median (*E*/*E*_0_)^2^, and (iii) minimum (*E*/*E*_0_)^2^ of the model. While these parameters have been optimized for single-width gratings, dual-width gratings can achieve even higher (*E*/*E*_0_)^2^ values.

### 3.2. Dual-Width Grating Optimization

Having determined the substrate thickness and Au width for maximizing optical enhancement in the single-width grating case, the dual-width geometry was then analyzed. At a constant *t*_SiO2_ of 330 nm, each wire width was swept from 20 to 500 nm by 10 nm increments. Figure 3 shows results from this calculation. Optical enhancement distributions in the gap are shown in Figure 3a (i)–(iv) for different structure width combinations. Depicted are the following cases: (i) *w*_1_ = 20 nm and *w*_2_ = 390 nm, (ii) *w*_1_ = *w*_2_ = 470 nm, (iii) *w*_1_ = *w*_2_ = 40 nm, and (iv) *w*_1_ = 410 nm and *w*_2_ = 70 nm. The gap region clearly shows changes to the local (*E*/*E*_0_)^2^ values for different dual-width situations. The average enhancement in the hot spot for a particular geometry is given by each data point of Figure 3b,c.

The color map in Figure 3b shows the average enhancement value at each width combination, with the dashed blue diagonal line corresponding to the single-width case (where *w*_1_ = *w*_2_). This blue line is the same as the horizontal blue line in Figure 2b. Along the gray negatively-sloped dashed line, all width combinations result in a constant period of *P* = 490 nm. In fact, any line drawn on this plot with a slope of −1 has a specific constant period, calculated (as defined) in Section 2. It is interesting to note that the geometries displaying peaks in enhancement tend to lie along specific lines of constant period on the color plot. From previous work, the locations of these peak enhancement periods depend on the incident wavelength [71]. They shift towards shorter periods for shorter wavelengths and towards larger width combinations for longer wavelengths, since the period is coupling with the incident light to create strong plasmonic grating effects. Points (i)–(iv) in Figure 3b correspond to the images in Figure 3a with the same labels. What is also interesting is that the greatest enhancement occurs not for this more traditional standard single-width grating geometry, but for a grating containing two different structure widths within each period. Over this range, the peak enhancement occurs when *w*_1_ = 410 nm and *w*_2_ = 70 nm, the case shown in Figure 3a (iv), indicating that a dual width geometry is superior to the traditional single-width plasmonic grating structures for increasing optical signals in sensing devices. 

The plot in Figure 3c shows the enhancement versus *w*_1_ from 20 to 500 nm along the blue and green dashed lines in Figure 3b, where blue is single width design (*w*_2_ = *w*_1_) and green is the dual width geometry, with one width held constant (*w*_2_ = 70 nm). While for small *w*_2_ values the optimal width is larger for the singe-width case, for larger widths *w*_2_ near 400 nm, the dual-width design demonstrates more than double the peak enhancement value compared to that of the single-width geometry, with the dual-width having the greatest overall enhancement value. Optimizing the oxide thickness for the dual-width design can improve this SERS sensor even further.

### 3.3. Coupled Optimization

**Figure 4:**
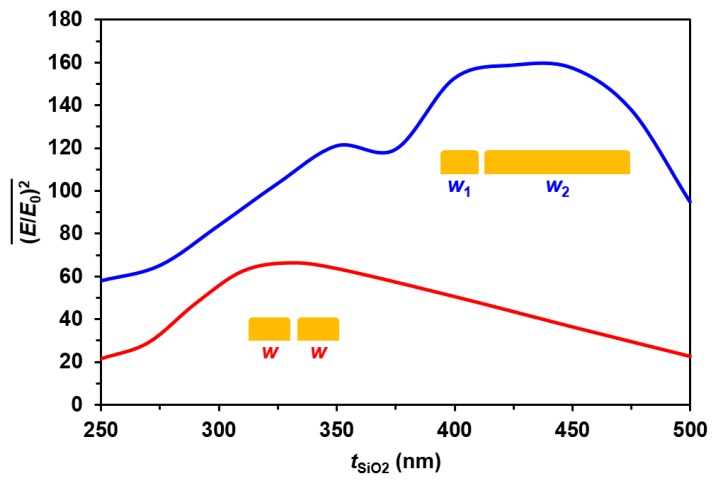
Calculated average enhancement versus *t*_SiO2_. The blue line shows the values at the optimal dual-width combination, averaging *w*_1_ = 72 nm and *w*_2_ = 409 nm, for each oxide thickness. The red line displays values at the optimal single-width value of *w* = 40 nm.

Lastly, *t*_SiO2_, *w*_1_, and *w*_2_ were optimized together. The width combination for maximum enhancement was determined for *t*_SiO2_ from 250 to 500 nm. The dual width thickness optimization peak is compared to the single-width peak, as a function of oxide thickness in Figure 4. The plot in blue shows the average gap field enhancement versus *t*_SiO2_ at the optimal width combinations, determined in Figure 3: *w*_1_ = 410 nm and *w*_2_ = 70 nm. The red plot in Figure 4 takes values from Figure 2 for the optimal single-width grating, where *w* = 40 nm, over the same oxide thickness range. This demonstrates the greater than 200% increase in enhancement that can theoretically be obtained by using a dual-width grating versus a single-width, both at optimal geometries.

The peak in enhancement is seen at *t*_SiO2_ = 425 nm for the dual-width grating design. At each oxide thickness, each dual width was swept to make sure that changing the oxide thickness did not alter the optimized dual width. The optimized widths did change slightly, but not by much. The peak values consistently occur for wire widths within ~10 nm of the average values, regardless of the substrate thickness. Across this range of thicknesses, the enhancement maxima occur at average widths *w*_1_ = 72 ± 3.8 nm and *w*_2_ = 409 ± 11 nm. Overall, coupling these structure widths with a SiO_2_ thickness of 425 nm will produce the greatest enhancement across this range of geometry values. For the same *w*_1_ and *w*_2_ combination with no oxide layer atop the substrate, the average value for (*E*/*E*_0_)^2^ within the gap region was near 1.

## 4. Discussion

The periodic nature of the average enhancement as a function of oxide thickness is due to thin-film interference within the dielectric. Therefore, when designing plasmonic SERS sensors to have the highest sensitivity of detection, choosing a substrate with optimal oxide thickness can improve the optical enhancement in the device by 200–300%.

In the case of the plasmonic grating on a varying *t*_SiO2_, the thin film interference effect for an incident light of constant wavelength and angle causes a constructive and destructive combination of the enhanced light. The interference at the gap region is between the incident light and that scattered from the plasmonic structures. Therefore, one must not simply choose a traditional quarter-wave thickness, as this may not always be the optimal oxide thickness for a particular device. This work demonstrates the need for careful modeling to engineer this ideal oxide thickness.

As has been previously demonstrated for plasmonic grating-on-GaAs photodetector applications, the dual-width grating geometry is theoretically capable of producing greater enhancement than a single-width alternative [71]. This is due to the dual-width geometry supporting hybrid plasmonic modes, which resonate with both an ideal grating period and an ideal metal structure width, simultaneously. For single-width gratings, the period may not be kept constant if the *w* value is varied. Due to these differences in tunability, a dual-width grating, optimized for a given substrate oxide thickness, can produce over 200% the enhancement given by a similarly optimized single-width grating. This additional enhancement can be more than 800% greater when comparing an ideal dual-width combination to a standard grating that has not been optimized for the substrate thickness. Therefore, it is necessary to simultaneously design the plasmonic structures and the underlying dielectric substrate thickness to produce the overall greatest enhancement factor for a given wavelength of incident light. The nanomasking fabrication process for creating nanogap gratings allows for this tunability and, therefore, holds promise for the highly tunable production of biosensor devices.

Other groups have demonstrated devices with high hot spot densities [80,81]. The optimal design in the present work still contains multiple hot spots per diffraction-limited laser beam spot. This eliminates the need to precisely align the probe beam to an individual nanostructure, unlike for a previously reported self-aligned nanogap SERS sensor design [82]. Instead, at least two hot spot regions are excited on the sensor substrate within a 1 micron excitation laser spot, regardless of the beam position. Thus, scanning the beam across the sample will cause enhanced scattering for any analyte molecules located in hot spot regions. Though other works may contain higher hot spot densities than the currently reported dual-width grating design [83,84,85], a dual-width grid design—which also makes use of the nanomasking fabrication technique [62]—can be implemented to greatly increase the number of gaps per area. The optimization of this more complex geometry will require further modeling.

## 5. Conclusions 

This study optimized geometries in plasmonic nanostructures to improve optical enhancement (*E*/*E*_0_)^2^ for SERS biosensing. Computational models were built to simulate Au structures, adhered to a SiO_2_/Si substrate by a 1 nm thick Ti layer. Holding constant the structure spacing at 5 nm, Ti thickness at 1 nm, and Au thickness at 15 nm, the metal structure width (*w*) and SiO_2_ thickness (*t*_SiO2_) were optimized for both single and dual-width gratings. The single-width grating displays an enhancement response that is periodic as *t*_SiO2_ increases, due to thin-film interference. A dual-width gratings design can generate more than double the (*E*/*E*_0_)^2^ value in the nanogap than that observed for single-width gratings at their greatest peaks in enhancement. This supports previous work on the use of such dual-width plasmonic gratings to enhance GaAs photodetectors [71]. For single-width gratings, the optimized geometry includes *w* = 40 nm and *t*_SiO2_ = 330 nm, while for dual-width gratings, the combination of *w*_1_ = 72 nm, *w*_2_ = 409 nm, and *t*_SiO2_ = 425 nm produces the greatest enhancement. The final optimized dual-width structure and SiO_2_ thickness combination gives enhancement values that are up to 800% greater than those exhibited by structures with non-ideal single-width gratings and oxide thicknesses. This 800% enhancement factor increase compares the best dual-width geometry combination to the worst single-width value, while a 200% increase is measured when comparing the best of the dual- and single-width cases.

This computational work will assist future research by guiding the fabrication of SERS biosensing devices prior to experimental characterization. Future work is necessary to investigate a wider range of values for the variables tested herein, and to test these results experimentally. Every new insight into these types of dual-width grating sensors moves them closer to a feasible device for fabrication and potential use in the biosensing industry.

## References

[1] Jain A., Liu R., Ramani B., Arauz E., Ishitsuka Y., Ragunathan K., Park J., Chen J., Xiang Y.K., Ha T. (2011). Probing cellular protein complexes using single-molecule pull-down. Nature.

[2] Cui X., Tawa K., Kintaka K., Nishii J. (2010). Enhanced Fluorescence Microscopic Imaging by Plasmonic Nanostructures: From a 1D Grating to a 2D Nanohole Array. Adv. Funct. Mater..

[3] Wertz E., Isaacoff B.P., Flynn J.D., Biteen J.S. (2015). Single-Molecule Super-Resolution Microscopy Reveals How Light Couples to a Plasmonic Nanoantenna on the Nanometer Scale. Nano Lett..

[4] Sone J., Fujita J., Ochiai Y., Manako S., Matsui S., Nomura E., Baba T., Kawaura H., Sakamoto T., Chen C.D. (1999). Nanofabrication toward sub-10 nm and its application to novel nanodevices. Nanotechnology.

[5] Chen Y., Pépin A. (2001). Nanofabrication: Conventional and nonconventional methods. Electrophoresis.

[6] Biswas A., Bayer I.S., Biris A.S., Wang T., Dervishi E., Faupel F. (2012). Advances in top-down and bottom-up surface nanofabrication: Techniques, applications & future prospects. Adv. Colloid Interface Sci..

[7] Moerner W.E. (2002). A Dozen Years of Single-Molecule Spectroscopy in Physics, Chemistry, and Biophysics. J. Phys. Chem. B.

[8] Beuwer M.A., Prins M.W.J., Zijlstra P. (2015). Stochastic Protein Interactions Monitored by Hundreds of Single-Molecule Plasmonic Biosensors. Nano Lett..

[9] Arroyo J.O., Kukura P. (2016). Non-fluorescent schemes for single-molecule detection, imaging and spectroscopy. Nat. Photon..

[10] Lecarme O., Sun Q., Ueno K., Misawa H. (2014). Robust and Versatile Light Absorption at Near-Infrared Wavelengths by Plasmonic Aluminum Nanorods. ACS Photon..

[11] Chen Y., Li Z., Xiang Q., Wang Y., Zhang Z., Duan H. (2015). Reliable fabrication of plasmonic nanostructures without an adhesion layer using dry lift-off. Nanotechnology.

[12] Cai H., Wu Y., Dai Y., Pan N., Tian Y., Luo Y., Wang X. (2016). Wafer scale fabrication of highly dense and uniform array of sub-5 nm nanogaps for surface enhanced Raman scatting substrates. Opt. Express.

[13] Boukobza E., Sonnenfeld A., Haran G. (2001). Immobilization in Surface-Tethered Lipid Vesicles as a New Tool for Single Biomolecule Spectroscopy. J. Phys. Chem. B.

[14] Kane R.S., Stroock A.D. (2007). Nanobiotechnology: Protein-Nanomaterial Interactions. Biotechnol. Prog..

[15] Bantz K.C., Meyer A.F., Wittenberg N.J., Im H., Kurtuluş Ö., Lee S.H., Lindquist N.C., Oh S.-H., Haynes C.L. (2011). Recent progress in SERS biosensing. Phys. Chem. Chem. Phys..

[16] Campos L.A., Liu J., Wang X., Ramanathan R., English D.S., Muñoz V. (2011). A photoprotection strategy for microsecond-resolution single-molecule fluorescence spectroscopy. Nat. Method..

[17] Chung H.S., McHale K., Louis J.M., Eaton W.A. (2012). Single-Molecule Fluorescence Experiments Determine Protein Folding Transition Path Times. Science.

[18] Fu B., Flynn J.D., Isaacoff B.P., Rowland D.J., Biteen J.S. (2015). Super-Resolving the Distance-Dependent Plasmon-Enhanced Fluorescence of Single Dye and Fluorescent Protein Molecules. J. Phys. Chem. C.

[19] Chen Y., Munechika K., Ginger D.S. (2007). Dependence of Fluorescence Intensity on the Spectral Overlap between Fluorophores and Plasmon Resonant Single Silver Nanoparticles. Nano Lett..

[20] Chen C., Stevens B., Kaur J., Cabral D., Liu H., Wang Y., Zhang H., Rosenblum G., Smilansky Z., Goldman Y.E. (2011). Single-Molecule Fluorescence Measurements of Ribosomal Translocation Dynamics. Mol. Cell.

[21] Chen P., Shen A., Zhou X., Hu J. (2011). Bio-Raman spectroscopy: A potential clinical analytical method assisting in disease diagnosis. Anal. Method.

[22] Peng P., Huang H., Hu A., Gerlich A.P., Zhou Y.N. (2012). Functionalization of silver nanowire surfaces with copper oxide for surface-enhanced Raman spectroscopic bio-sensing. J. Mater. Chem..

[23] Demirel M.C., Kao P., Malvadkar N., Wang H., Gong X., Poss M., Allara D.L. (2009). Bio-organism sensing via surface enhanced Raman spectroscopy on controlled metal/polymer nanostructured substrates. Biointerphases.

[24] Motz J.T., Hunter M., Galindo L.H., Gardecki J.A., Kramer J.R., Dasari R.R., Feld M.S. (2004). Optical Fiber Probe for Biomedical Raman Spectroscopy. Appl. Opt..

[25] Zhang X., Shah N.C., Van Duyne R.P. (2006). Sensitive and selective chem/bio sensing based on surface-enhanced Raman spectroscopy (SERS). Vib. Spectrosc..

[26] Sharma S.K., Misra A.K., Sharma B. (2005). Portable remote Raman system for monitoring hydrocarbon, gas hydrates and explosives in the environment. Spectrochim. Acta Mol. Biomol. Spectrosc..

[27] Hargreaves M.D., Page K., Munshi T., Tomsett R., Lynch G., Edwards H.G.M. (2008). Analysis of seized drugs using portable Raman spectroscopy in an airport environment—A proof of principle study. J. Raman Spectrosc..

[28] Izake E.L. (2010). Forensic and homeland security applications of modern portable Raman spectroscopy. Forensic Sci. Int..

[29] Chi Z., Asher S.A. (1998). UV Raman Determination of the Environment and Solvent Exposure of Tyr and Trp Residues. J. Phys. Chem. B.

[30] Raman Suri C., Boro R., Nangia Y., Gandhi S., Sharma P., Wangoo N., Rajesh K., Shekhawat G.S. (2009). Immunoanalytical techniques for analyzing pesticides in the environment. TrAC Trends Anal. Chem..

[31] Tyler S.W., Selker J.S., Hausner M.B., Hatch C.E., Torgersen T., Thodal C.E., Schladow S.G. (2009). Environmental temperature sensing using Raman spectra DTS fiber-optic methods. Water Resour. Res..

[32] Halvorson R.A., Vikesland P.J. (2010). Surface-Enhanced Raman Spectroscopy (SERS) for Environmental Analyses. Environ. Sci. Technol..

[33] Álvarez-Puebla R.A., Liz-Marzán L.M. (2010). Environmental applications of plasmon assisted Raman scattering. Energy Environ. Sci..

[34] Moore D.S., Scharff R.J. (2009). Portable Raman explosives detection. Anal. Bioanal. Chem..

[35] Pallaoro A., Hoonejani M.R., Braun G.B., Meinhart C., Moskovits M. (2013). Combined surface-enhanced Raman spectroscopy biotags and microfluidic platform for quantitative ratiometric discrimination between noncancerous and cancerous cells in flow. J. Nanophoton..

[36] Huang R., Han S., Li X. (2013). (Sheryl) Detection of tobacco-related biomarkers in urine samples by surface-enhanced Raman spectroscopy coupled with thin-layer chromatography. Anal. Bioanal. Chem..

[37] Maiwald M., Müller A., Sumpf B., Tränkle G. (2016). A portable shifted excitation Raman difference spectroscopy system: Device and field demonstration. J. Raman Spectrosc..

[38] George T.F., Markel V.A. (2001). Optics of Nanostructured Materials. Meas. Sci. Technol..

[39] Maier S.A., Atwater H.A. (2005). Plasmonics: Localization and guiding of electromagnetic energy in metal/dielectric structures. J. Appl. Phys..

[40] Ahn S., Rourke D., Park W. (2016). Plasmonic nanostructures for organic photovoltaic devices. J. Opt..

[41] Kuznetsov A.I., Miroshnichenko A.E., Brongersma M.L., Kivshar Y.S., Luk’yanchuk B. (2016). Optically resonant dielectric nanostructures. Science.

[42] Schuller J.A., Barnard E.S., Cai W., Jun Y.C., White J.S., Brongersma M.L. (2010). Plasmonics for extreme light concentration and manipulation. Nat. Mater..

[43] Lee B., Lee I.M., Kim S., Oh D.H., Hesselink L. (2010). Review on subwavelength confinement of light with plasmonics. J. Mod. Opt..

[44] Huang Y., Fang Y., Zhang Z., Zhu L., Sun M. (2014). Nanowire-supported plasmonic waveguide for remote excitation of surface-enhanced Raman scattering. Light Sci. Appl..

[45] Abdulrahman R.B., Cansizoglu H., Cansizoglu M.F., Herzog J.B., Karabacak T. (2015). Enhanced light trapping and plasmonic properties of aluminum nanorods fabricated by glancing angle deposition. J. Vac. Sci. Technol. A.

[46] Ward D.R., Hüser F., Pauly F., Cuevas J.C., Natelson D. (2010). Optical rectification and field enhancement in a plasmonic nanogap. Nat. Nanotechnol..

[47] Lin J., Oh S.H., Nguyen H.M., Reitich F. (2014). Field enhancement and saturation of millimeter waves inside a metallic nanogap. Opt. Express.

[48] Edwards A.P., Adawi A.M. (2014). Plasmonic nanogaps for broadband and large spontaneous emission rate enhancement. J. Appl. Phys..

[49] Chen X., Ciracì C., Smith D.R., Oh S.H. (2015). Nanoga-Enhanced Infrared Spectroscopy with Template-Stripped Wafer-Scale Arrays of Buried Plasmonic Cavities. Nano Lett..

[50] Park H.R., Chen X., Nguyen N.C., Peraire J., Oh S.-H. (2015). Nanogap-Enhanced Terahertz Sensing of 1 nm Thick (λ/106) Dielectric Films. ACS Photon..

[51] Zhu W., Esteban R., Borisov A.G., Baumberg J.J., Nordlander P., Lezec H.J., Aizpurua J., Crozier K.B. (2016). Quantum mechanical effects in plasmonic structures with subnanometre gaps. Nat. Commun..

[52] Kneipp J., Kneipp H., Kneipp K. (2008). SERS—A single-molecule and nanoscale tool for bioanalytics. Chem. Soc. Rev..

[53] Etchegoin P.G., Le Ru E.C., Schlücker S. (2010). Basic Electromagnetic Theory of SERS. Surface Enhanced Raman Spectroscopy.

[54] Wang A.X., Kong X. (2015). Review of Recent Progress of Plasmonic Materials and Nano-Structures for Surface-Enhanced Raman Scattering. Materials.

[55] Etchegoin P.G., Le Ru E.C. (2008). A perspective on single molecule SERS: Current status and future challenges. Phys. Chem. Chem. Phys..

[56] Tokel O., Inci F., Demirci U. (2014). Advances in Plasmonic Technologies for Point of Care Applications. Chem. Rev..

[57] Long J., Yang T. (2016). Observation of Single Molecule Dynamic Behaviors with SERS: Desorption and Conformation Switching. Proceedings of the Conference on Lasers and Electro-Optics (2016).

[58] Theiss J., Pavaskar P., Echternach P.M., Muller R.E., Cronin S.B. (2010). Plasmonic Nanoparticle Arrays with Nanometer Separation for High-Performance SERS Substrates. Nano Lett..

[59] Dinish U.S., Yaw F.C., Agarwal A., Olivo M. (2011). Development of highly reproducible nanogap SERS substrates: Comparative performance analysis and its application for glucose sensing. Biosens. Bioelectron..

[60] Chung T., Lee S.-Y., Song E.Y., Chun H., Lee B. (2011). Plasmonic Nanostructures for Nano-Scale Bio-Sensing. Sensors.

[61] Kho K.W., Dinish U.S., Kumar A., Olivo M. (2012). Frequency Shifts in SERS for Biosensing. ACS Nano.

[62] Bauman S.J., Novak E.C., Debu D.T., Natelson D., Herzog J.B. (2015). Fabrication of Sub-Lithography-Limited Structures via Nanomasking Technique for Plasmonic Enhancement Applications. IEEE Trans. Nanotechnol..

[63] Byun K.M., Kim S.J., Kim D. (2007). Grating-coupled transmission-type surface plasmon resonance sensors based on dielectric and metallic gratings. Appl. Opt..

[64] Dhawan A., Canva M., Vo-Dinh T. (2011). Narrow groove plasmonic nano-gratings for surface plasmon resonance sensing. Opt. Express.

[65] Chen B., Wood A., Pathak A., Mathai J., Bok S., Zheng H., Hamm S., Basuray S., Grant S., Gangopadhyay K. (2016). Plasmonic gratings with nano-protrusions made by glancing angle deposition for single-molecule super-resolution imaging. Nanoscale.

[66] Siegfried T., Ekinci Y., Solak H.H., Martin O.J.F., Sigg H. (2011). Fabrication of sub-10 nm gap arrays over large areas for plasmonic sensors. Appl. Phys. Lett..

[67] Feng J., Okamoto T., Kawata S. (2005). Enhancement of electroluminescence through a two-dimensional corrugated metal film by grating-induced surface-plasmon cross coupling. Opt. Lett..

[68] López-Tejeira F., Rodrigo S.G., Martín-Moreno L., García-Vidal F.J., Devaux E., Ebbesen T.W., Krenn J.R., Radko I.P., Bozhevolnyi S.I., González M.U. (2007). Efficient unidirectional nanoslit couplers for surface plasmons. Nat. Phys..

[69] Jiao Y., Liu L.H., Hsu P.F. (2013). Widening Absorption Band of Grating Structure With Complex Dual-Groove Grating. J. Heat Transf..

[70] Estakhri N.M., Neder V., Knight M.W., Polman A., Alù A. (2017). Visible Light, Wide-Angle Graded Metasurface for Back Reflection. ACS Photon..

[71] Darweesh A.A., Bauman S.J., Herzog J.B. (2016). Improved optical enhancement using double-width plasmonic gratings with nanogaps. Photon. Res..

[72] Aizpurua J., Bryant G.W., Richter L.J., García de Abajo F.J., Kelley B.K., Mallouk T. (2005). Optical properties of coupled metallic nanorods for field-enhanced spectroscopy. Phys. Rev. B.

[73] Grimault A.-S., Vial A., Grand J., Lamy de La Chapelle M. (2008). Modelling of the near-field of metallic nanoparticle gratings: Localized surface plasmon resonance and SERS applications. J. Microsc..

[74] Colas F.J., Cottat M., Gillibert R., Guillot N., Djaker N., Lidgi-Guigui N., Toury T., Barchiesi D., Toma A., Di Fabrizio E. (2016). Red-Shift Effects in Surface Enhanced Raman Spectroscopy: Spectral or Intensity Dependence of the Near-Field?. J. Phys. Chem. C.

[75] Bryche J.-F., Gillibert R., Barbillon G., Gogol P., Moreau J., Lamy de la Chapelle M., Bartenlian B., Canva M. (2016). Plasmonic Enhancement by a Continuous Gold Underlayer: Application to SERS Sensing. Plasmonics.

[B76-sensors-17-01530] 76.COMSOL Multiphysics^®^, v. 5.0. www.comsol.com. COMSOL AB, Stockholm, Sweden.

[77] Debu D.T., Ghosh P.K., French D., Herzog J.B. (2017). Surface plasmon damping effects due to Ti adhesion layer in individual gold nanodisks. Opt. Mater. Express.

[78] Ghosh P.K., Debu D.T., French D.A., Herzog J.B. (2017). Calculated thickness dependent plasmonic properties of gold nanobars in the visible to near-infrared light regime. PLoS ONE.

[79] Brawley Z.T., Bauman S.J., Abbey G.P., Darweesh A.A., Nusir A.I., Manasreh O., Herzog J.B. (2017). Modeling and optimization of Au-GaAs plasmonic nanoslit array structures for enhanced near-infrared photodetector applications. J. Nanophoton..

[80] Kessentini S., Barchiesi D., D’Andrea C., Toma A., Guillot N., Di Fabrizio E., Fazio B., Maragó O.M., Gucciardi P.G., Lamy de la Chapelle M. (2014). Gold Dimer Nanoantenna with Slanted Gap for Tunable LSPR and Improved SERS. J. Phys. Chem. C.

[81] D’Andrea C., Fazio B., Gucciardi P.G., Giordano M.C., Martella C., Chiappe D., Toma A., Buatier de Mongeot F., Tantussi F., Vasanthakumar P. (2014). SERS Enhancement and Field Confinement in Nanosensors Based on Self-Organized Gold Nanowires Produced by Ion-Beam Sputtering. J. Phys. Chem. C.

[82] Herzog J.B., Knight M.W., Li Y., Evans K.M., Halas N.J., Natelson D. (2013). Dark Plasmons in Hot Spot Generation and Polarization in Interelectrode Nanoscale Junctions. Nano Lett..

[83] Le Ru E.C., Etchegoin P.G., Grand J., Félidj N., Aubard J., Lévi G., Hohenau A., Krenn J.R. (2008). Surface enhanced Raman spectroscopy on nanolithography-prepared substrates. Curr. Appl. Phys..

[84] Caldwell J.D., Glembocki O., Bezares F.J., Bassim N.D., Rendell R.W., Feygelson M., Ukaegbu M., Kasica R., Shirey L., Hosten C. (2011). Plasmonic Nanopillar Arrays for Large-Area, High-Enhancement Surface-Enhanced Raman Scattering Sensors. ACS Nano.

[85] Cottat M., D’Andrea C., Yasukuni R., Malashikhina N., Grinyte R., Lidgi-Guigui N., Fazio B., Sutton A., Oudar O., Charnaux N. (2015). High Sensitivity, High Selectivity SERS Detection of MnSOD Using Optical Nanoantennas Functionalized with Aptamers. J. Phys. Chem. C.

